# Pattern analysis of laser-tattoo interactions for picosecond- and nanosecond-domain 1,064-nm neodymium-doped yttrium-aluminum-garnet lasers in tissue-mimicking phantom

**DOI:** 10.1038/s41598-017-01724-1

**Published:** 2017-05-08

**Authors:** Keun Jae Ahn, Zhenlong Zheng, Tae Rin Kwon, Beom Joon Kim, Hye Sun Lee, Sung Bin Cho

**Affiliations:** 1 Department of Science Education, Jeju National University, Jeju, Korea; 2 Department of Dermatology, Yanbian University Hospital, Yanji, China; 3 Department of Dermatology and Cutaneous Biology Research Center, International St. Mary’s Hospital, Catholic Kwandong University College of Medicine, Incheon, Korea; 4 Department of Dermatology, Chung-Ang University College of Medicine, Seoul, Korea; 5 Department of Medicine, Graduate School, Chung-Ang University, Seoul, Korea; 6 Biostatistics Collaboration Unit, Yonsei University College of Medicine, Seoul, Korea; 7 Kangskin Sillim Dermatology Clinic, Seoul, Korea

## Abstract

During laser treatment for tattoo removal, pigment chromophores absorb laser energy, resulting in fragmentation of the ink particles via selective photothermolysis. The present study aimed to outline macroscopic laser-tattoo interactions in tissue-mimicking (TM) phantoms treated with picosecond- and nanosecond-domain lasers. Additionally, high-speed cinematographs were captured to visualize time-dependent tattoo-tissue interactions, from laser irradiation to the formation of photothermal and photoacoustic injury zones (PIZs). In all experimental settings using the nanosecond or picosecond laser, tattoo pigments fragmented into coarse particles after a single laser pulse, and further disintegrated into smaller particles that dispersed toward the boundaries of PIZs after repetitive delivery of laser energy. Particles fractured by picosecond treatment were more evenly dispersed throughout PIZs than those fractured by nanosecond treatment. Additionally, picosecond-then-picosecond laser treatment (5-pass-picosecond treatment + 5-pass-picosecond treatment) induced greater disintegration of tattoo particles within PIZs than picosecond-then-nanosecond laser treatment (5-pass-picosecond treatment + 5-pass-nanosecond treatment). High-speed cinematography recorded the formation of PIZs after repeated reflection and propagation of acoustic waves over hundreds of microseconds to a few milliseconds. The present data may be of use in predicting three-dimensional laser-tattoo interactions and associated reactions in surrounding tissue.

## Introduction

Tattoo pigment chromophores absorb laser energy at particular wavelengths and pulse durations, thereby fragmenting into smaller particles, depending on their color and size, by selective photothermolysis^1, 2^. Upon absorption of laser energy, the chromophores explosively expand, generating acoustic waves that propagate to surrounding tissues, referred to as photoacoustic effects^3, 4^. Subsequently, the fractured tattoo particles are removed through transepidermal elimination, phagocytosis, and lymphatic drainage^1^.

Computational simulation study of laser-tissue interactions found photoacoustic effects to be the most salient mechanism of laser-assisted tattoo removal and that a shorter laser pulse results in more efficient breakdown of tattoo particles^5^. Q-switched lasers emit a laser beam with high peak power at an ultra-short nanosecond-pulse duration and have been widely used for tattoo removal^6^. Theoretically, however, lasers with a picosecond-pulse duration may be more advantageous to the selective breakdown of tattoo particles, since the sizes of tattoo pigment particles generally range from 10–100 nm and show a thermal relaxation time of 0.1–10 nsec^7–9^. Furthermore, *in vivo* and *in vitro* experiments suggest that, due to recent technological advances, picosecond-domain lasers are safe and effective for use in tattoo removal^7–12^.

Experimental settings for simulating laser-tattoo pigment interactions using a tattoo pigment-embedded tissue-mimicking (TM) phantom can exhibit various patterns of photothermal and photoacoustic injury zone (PIZ) formation for various treatment settings. Although TM phantoms are not identical to *in vivo* human skin, due to the lack of cellular components, adnexal structures, and a layered composition, PIZs in TM phantom have been deemed to accurately reflect macroscopic laser-tattoo interactions and surrounding tissue reactions immediately after laser treatment for tattoo removal. In the present comparison study, we analyzed and compared patterns of laser-tattoo interactions using picosecond- and nanosecond-domain neodymium-doped yttrium-aluminum-garnet (Nd:YAG) lasers on a tattoo pigment-embedded TM phantom. Additionally, time-dependent interactions, from laser irradiation to PIZ formation, in tissue surrounding the tattoo pigment were captured by high-speed cinematographs.

## Results

### Picosecond- and nanosecond-domain Nd:YAG laser treatment on tattoo ink embedded in TM phantom

**Figure 1:**
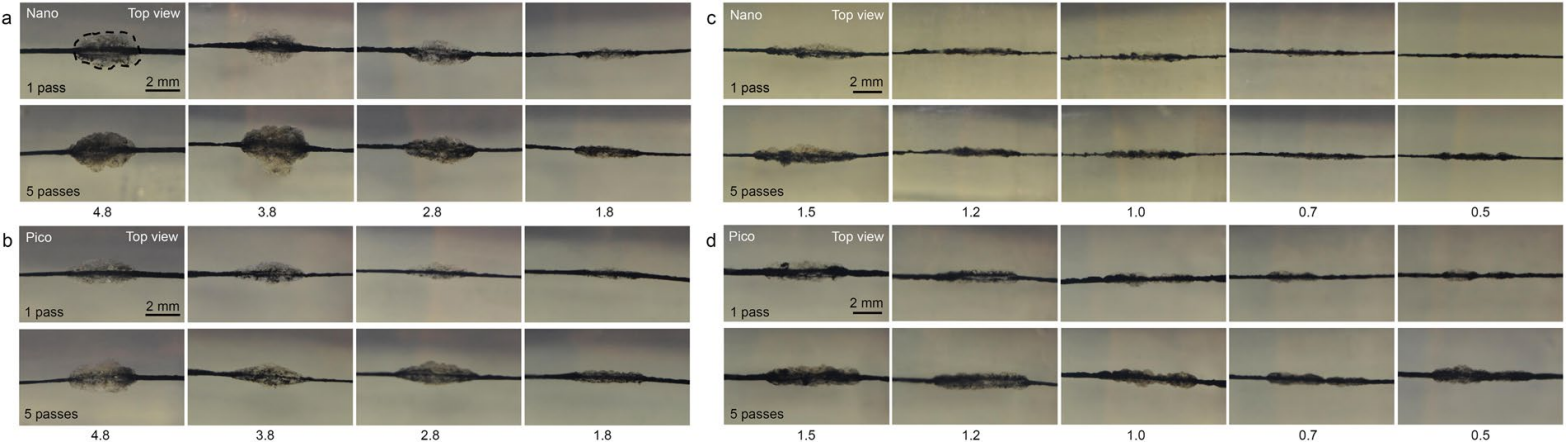
Picosecond- and nanosecond-domain neodymium-doped yttrium aluminum garnet (Nd:YAG) laser treatments on tattoo ink embedded in tissue-mimicking (TM) phantom. Both the (**a**) nanosecond- and (**b**) picosecond-domain Nd:YAG laser treatments generated cocoon-shaped or oval photothermal and photoacoustic injury zones (PIZ) at the laser fluences of 4.8 J/cm^2^, 3.8 J/cm^2^, 2.8 J/cm^2^, and 1.8 J/cm^2^ at a spot size of 4 mm. At laser fluences of 1.5 J/cm^2^, 1.2 J/cm^2^, 1.0 J/cm^2^, 0.7 J/cm^2^, and 0.5 J/cm^2^ and a spot size of 7 mm, cocoon-shaped PIZs formed after the (**c**) nanosecond- and (**d**) picosecond-domain Nd:YAG laser treatments. Top view; laser fluences are presented as J/cm^2^; Nano, nanosecond-domain laser; Pico, picosecond-domain laser. Dotted lines encircle a representative PIZ.

To analyze laser-tattoo interactions induced by treatment with picosecond- and nanosecond-domain Nd:YAG lasers, laser fluences of 1.8 J/cm^2^, 2.8 J/cm^2^, 3.8 J/cm^2^, and 4.8 J/cm^2^ were delivered to tattoo ink-embedded TM phantoms at a spot size of 4 mm. Both the picosecond- and nanosecond-domain Nd:YAG laser treatments generated cocoon-shaped or oval PIZs, and the higher the laser fluence, the bigger the sizes of the PIZs were (Fig. 1a,b). After five passes with the lasers, the sizes of the PIZs increased and the margins thereof became more obvious, compared to a single pass. Meanwhile, in all treatment settings, the widths of the PIZs were larger than the heights thereof, which was likely correlated with the beam sizes.

Additional experiments using the picosecond- and nanosecond-domain Nd:YAG lasers were performed at the laser fluences of 0.5 J/cm^2^, 0.7 J/cm^2^, 1.0 J/cm^2^, 1.2 J/cm^2^, and 1.5 J/cm^2^ and a spot size of 7 mm. Again, both the picosecond- and nanosecond-domain Nd:YAG laser treatments induced the formation of cocoon-shaped PIZs, and the higher the laser fluence, the larger the PIZs were (Fig. 1c,d). After five passes, the sizes of the PIZs increased, and the margins of the PIZs became more pronounced, compared to a single pass. Picosecond-domain Nd:YAG laser treatment showed remarkable laser fluence-dependent differences in the photoacoustic effects on tattoo particles in TM phantoms, even at the laser fluences of 0.5 J/cm^2^, 0.7 J/cm^2^, and 1.0 J/cm^2^, compared to nanosecond-domain laser treatment at the same settings.

Tattoo pigment fragmented into coarse particles after a single laser pulse and were further disintegrated into smaller particles that scattered toward the boundaries of PIZs after repeated delivery of laser pulses in all experimental settings. The disintegrated particles created during picosecond treatment were more evenly distributed throughout PIZs than the particles generated during nanosecond treatment; PIZs created by nanosecond treatment appeared to be blotchy and uneven. The patterns of particle destruction and redistribution were more evident after five passes of laser treatment. Also, core pigments of the baseline tattoos were more markedly fragmented and dispersed to the peripheral areas of PIZs in picosecond treatment than in nanosecond treatment (Supplementary Fig. 1a–d). Additionally, cavitation bubbles were more readily apparent after treatment with the picosecond laser, compared to treatment with the nanosecond laser.

### Combined treatment with picosecond- and nanosecond-domain Nd:YAG lasers on tattoo ink embedded in TM phantom

**Figure 2:**
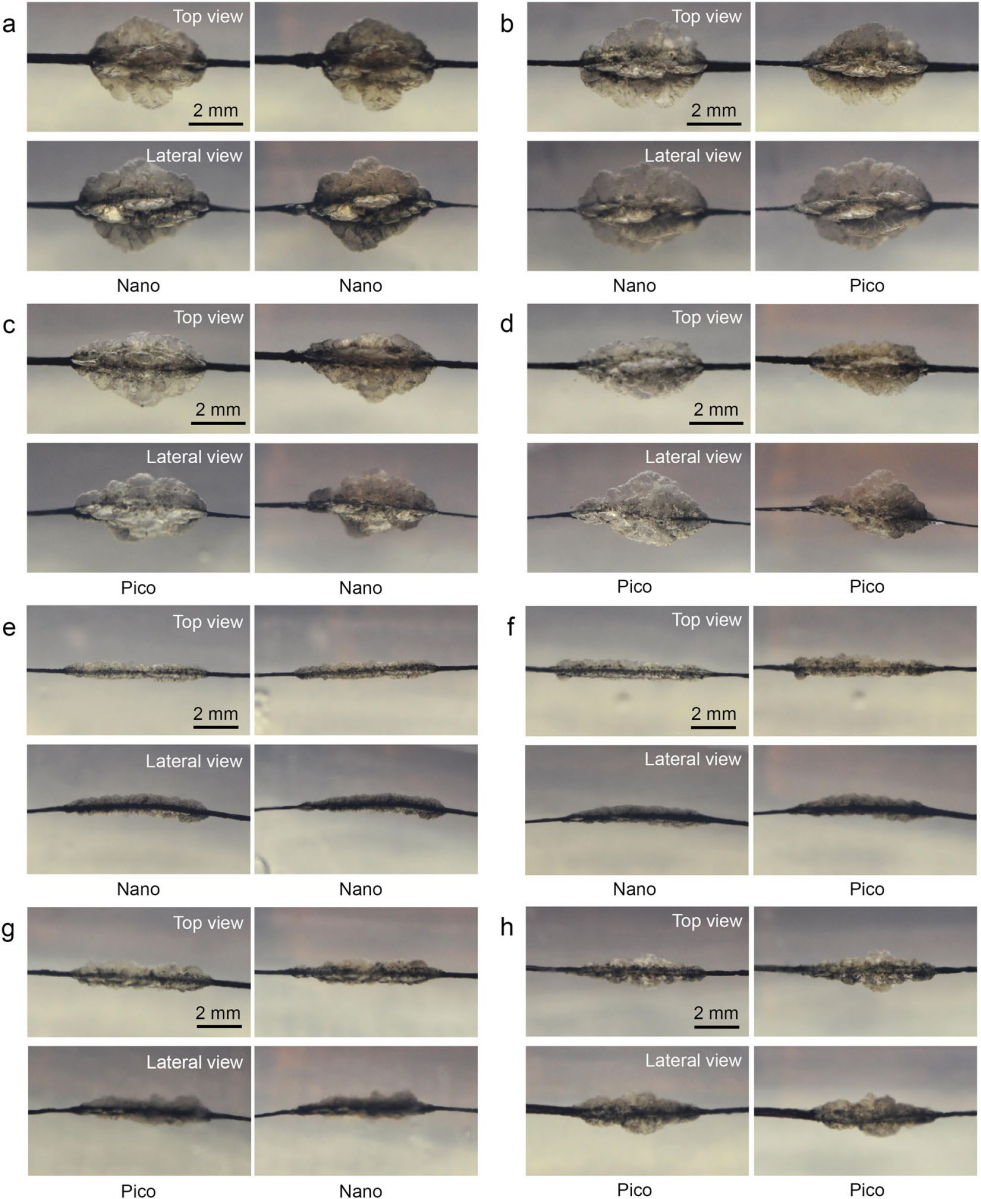
Combination of picosecond- and nanosecond-domain Nd:YAG laser treatment on tattoo ink embedded in TM phantom. Five passes of laser treatment using each of the picosecond- and nanosecond-domain Nd:YAG lasers were initially performed to fracture the ink particles. Then, an additional five passes with either the nanosecond- or picosecond-domain Nd:YAG laser were delivered. (**a**) Nanosecond-then-nanosecond, (**b**) nanosecond-then-picosecond, (**c**) picosecond-then-nanosecond, and (**d**) picosecond-then-picosecond at the experimental settings of a 4-mm spot size and a laser fluence of 4.8 J/cm^2^. (**e**) Nanosecond-then-nanosecond, (**f**) nanosecond-then-picosecond, (**g**) picosecond-then-nanosecond, and (**h**) picosecond-then-picosecond at the experimental settings of a 7-mm spot size and a laser fluence of 1.5 J/cm^2^. Top and lateral views; Nano, nanosecond-domain laser; Pico, picosecond-domain laser.

To evaluate the effects of further treatment with picosecond and nanosecond Nd:YAG lasers on fractured tattoo particles, five passes of laser treatment with the picosecond- or nanosecond-domain Nd:YAG laser were initially applied to the TM phantoms to disintegrate the ink particles. Then, an additional five passes with the nanosecond- or picosecond-domain Nd:YAG laser were delivered, and the morphological results were analyzed. At a 4-mm spot size, the initial treatments with the nanosecond and picosecond lasers generated oval PIZs containing fractured tattoo particles (Fig. 2a–d; Supplementary Fig. 2a–d). After delivering an additional five passes of nanosecond or picosecond laser treatment, the disintegrated tattoo particles were further destroyed into smaller fragments and widely distributed throughout the PIZs. The morphologic differences in PIZs between the nanosecond-then-nanosecond and nanosecond-then-picosecond laser treatments were not remarkable. Importantly, however, picosecond-then-picosecond laser treatment induced greater disintegration and dispersion of core pigments of the baseline tattoos into peripheral areas within PIZs than picosecond-then-nanosecond laser treatment.

At a spot size of 7 mm, cocoon-shaped PIZs containing fractured tattoo particles were initially generated with the both picosecond and nanosecond lasers (Fig. 2e–h). Tattoo pigments in deeper parts of picosecond-treated phantoms were noticeably destroyed, and more even disintegration of ink particles was observed, compared to the nanosecond-treated phantoms. An additional five passes with either the nanosecond or picosecond laser further destroyed the ink into smaller particles, which spread across the PIZs. Laser-tattoo interactions were more remarkable for the nanosecond-then-picosecond treatment than the nanosecond-then-nanosecond treatment. Both the picosecond-then-nanosecond and the picosecond-then-picosecond TM phantoms exhibited greater destruction of particles into homogeneous fragments; this interaction was more readily apparent in TM phantoms treated with picosecond-then-picosecond treatment.

### High-speed cinematography of picosecond- and nanosecond-domain Nd:YAG laser treatment on tattoo ink embedded in TM phantom

**Figure 3:**
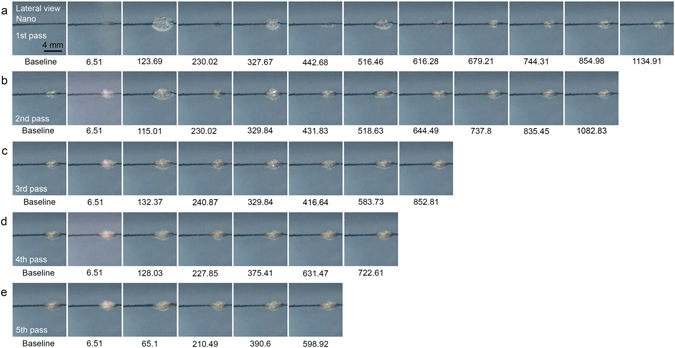
High-speed cinematography of nanosecond-domain Nd:YAG laser treatment on tattoo-embedded TM phantom. Captured frames exhibit the development of PIZs after repeated courses of acoustic wave reflection and propagation immediately after nanosecond-domain Nd:YAG laser treatment at the laser fluence of 4.8 J/cm^2^ and a spot size of 4 mm over five pulses at 5 Hz. Lateral view; times are presented as μsec; Nano, nanosecond-domain laser.

High-speed cinematographs were captured to visualize the effects of Nd:YAG laser treatment at ultra-short picosecond- and nanosecond-pulse durations on tattoo particles, from the initial laser irradiation to PIZ formation. Frames captured at 2.17 µsec and 5.34 µsec reflected strong flashes of light, and thus, those at 6.51 µsec were regarded as representing the earliest macroscopic laser-tattoo interactions. With the nanosecond-domain Nd:YAG laser, a fluence of 4.8 J/cm^2^ and a spot size of 4 mm induced maximum photoacoustic expansion of irradiated chromophores at 123.69 µsec, which was followed by minimum collapsing at 230.02 µsec, during the first pass of the laser over the TM phantom (Fig. 3, Supplementary video 1). Over five subsequent passes, the magnitude of the photoacoustic expansion and collapsing gradually decreased. PIZs presented no further remarkable morphologic changes beyond 1134.91 µsec. Upon delivering additional laser pulses, the number of expansion and collapsing sequences, as well as the magnitudes thereof, decreased.

**Figure 4:**
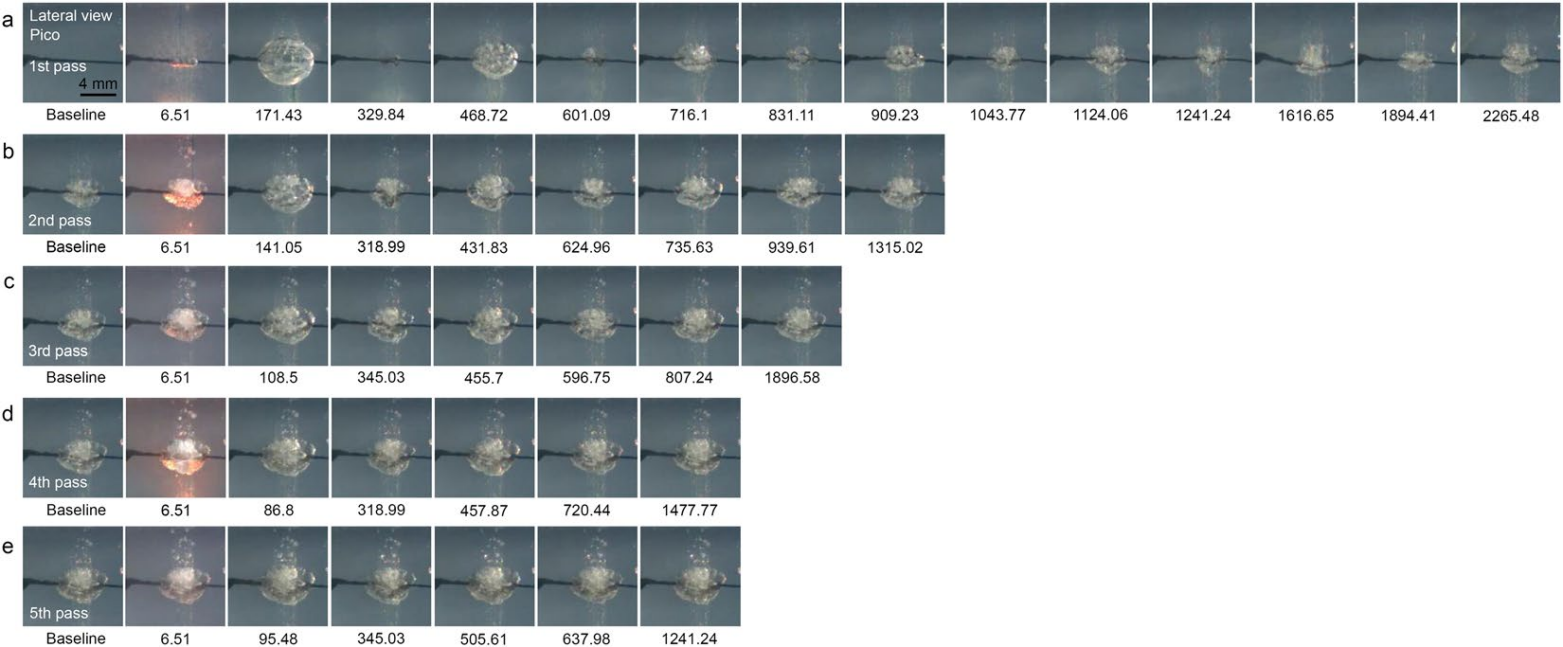
High-speed cinematography of picosecond-domain Nd:YAG laser treatments on tattoo-embedded TM phantom. Captured frames highlight the development of PIZs after repeated courses of acoustic wave reflection and propagation immediately after picosecond-domain Nd:YAG laser treatment at the laser fluence of 4.8 J/cm^2^ and a spot size of 4 mm over five pulses at 5 Hz. Lateral view; times are presented as μsec; Pico, picosecond-domain laser.

With the picosecond-domain Nd:YAG laser, a fluence of 4.8 J/cm^2^ and a spot size of 4 mm caused a red-hot appearance to form at the bottom of PIZs at 6.51 µsec, regardless of the number of laser passes. The maximum photoacoustic expansion after the first pass of the laser treatment over the TM phantom was recorded at 171.43 µsec and minimum collapsing was noted at 329.84 µsec (Fig. 4, Supplementary video 2). Expansive acoustic waves in the picosecond model were observed around 1616.65 µsec, and resulted in remarkable fluctuations in the tattoo in the TM phantom. The overall number of expansion and collapsing sequences was higher for the picosecond laser treatment than the nanosecond laser treatment. Otherwise, similar patterns of expansion and collapsing and variations in the magnitudes thereof were noted between the picosecond and nanosecond models.

**Figure 5:**
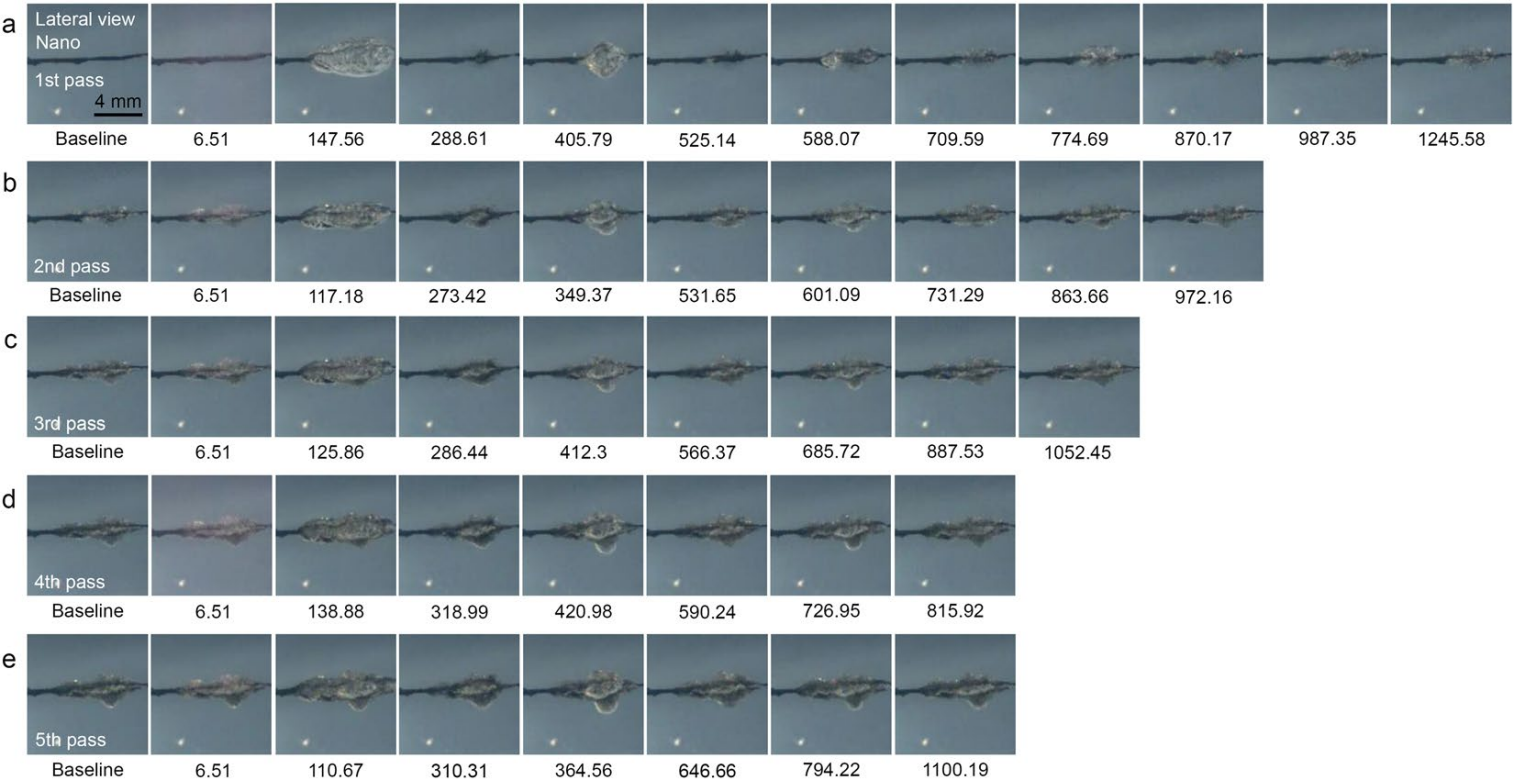
High-speed cinematography of nanosecond-domain Nd:YAG laser treatments on tattoo-embedded TM phantom. Captured frames present the development of PIZs after repeated courses of acoustic wave reflection and propagation immediately after nanosecond-domain Nd:YAG laser treatment at the laser fluence of 1.5 J/cm^2^ and a spot size of 7 mm over five pulses at 5 Hz. Lateral view; times are presented as μsec; Nano, nanosecond-domain laser.

**Figure 6:**
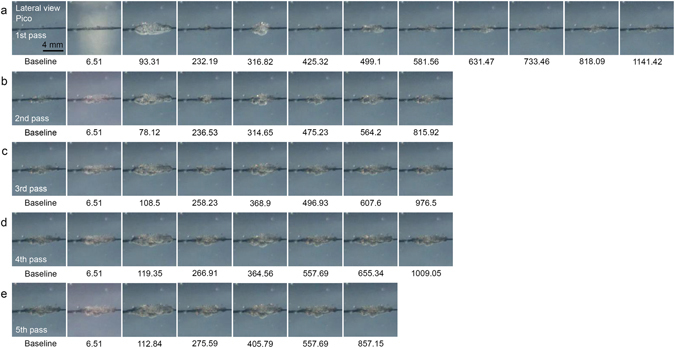
High-speed cinematography of picosecond-domain Nd:YAG laser treatment on tattooembedded TM phantom. Captured frames reflect the development of PIZs after repeated courses of acoustic wave reflection and propagation immediately after picosecond-domain Nd:YAG laser treatment at the laser fluence of 1.5 J/cm^2^ and a spot size of 7 mm over five pulses at 5 Hz. Lateral view; times are presented as μsec; Pico, picosecond-domain laser.

Meanwhile, in contrast to treatments using the picosecond and nanosecond lasers at a 4-mm spot size, use of the nanosecond laser at a fluence of 1.5 J/cm^2^ and a spot size of 7 mm failed to generate photoacoustic morphologic changes in the irradiated tattoo pigments at 6.51 µsec during the first pass of the laser treatments over the TM phantom (Fig. 5, Supplementary video 3). Maximum expansion was recorded at 147.56 µsec, followed by minimum collapsing at 288.61 µsec, during the first pass. Treatment with the picosecond-domain Nd:YAG laser at a fluence of 1.5 J/cm^2^ and a spot size of 7 mm also failed to induce photoacoustic morphologic changes at 6.51 µsec (Fig. 6, Supplementary video 4). Maximum expansion was recorded at 93.31 µsec, followed by minimum collapsing at 232.19 µsec, during the first pass of the picosecond treatment over the TM phantom. For the picosecond-domain laser, regardless of the number of laser passes, the appearance of a lingering flash could be observed around the irradiated tattoo pigments at 6.51 µsec. Destruction of core pigments of the baseline tattoos in the picosecond-treated phantoms was more readily apparent, compared to the nanosecond-treated phantoms.

**Figure 7:**
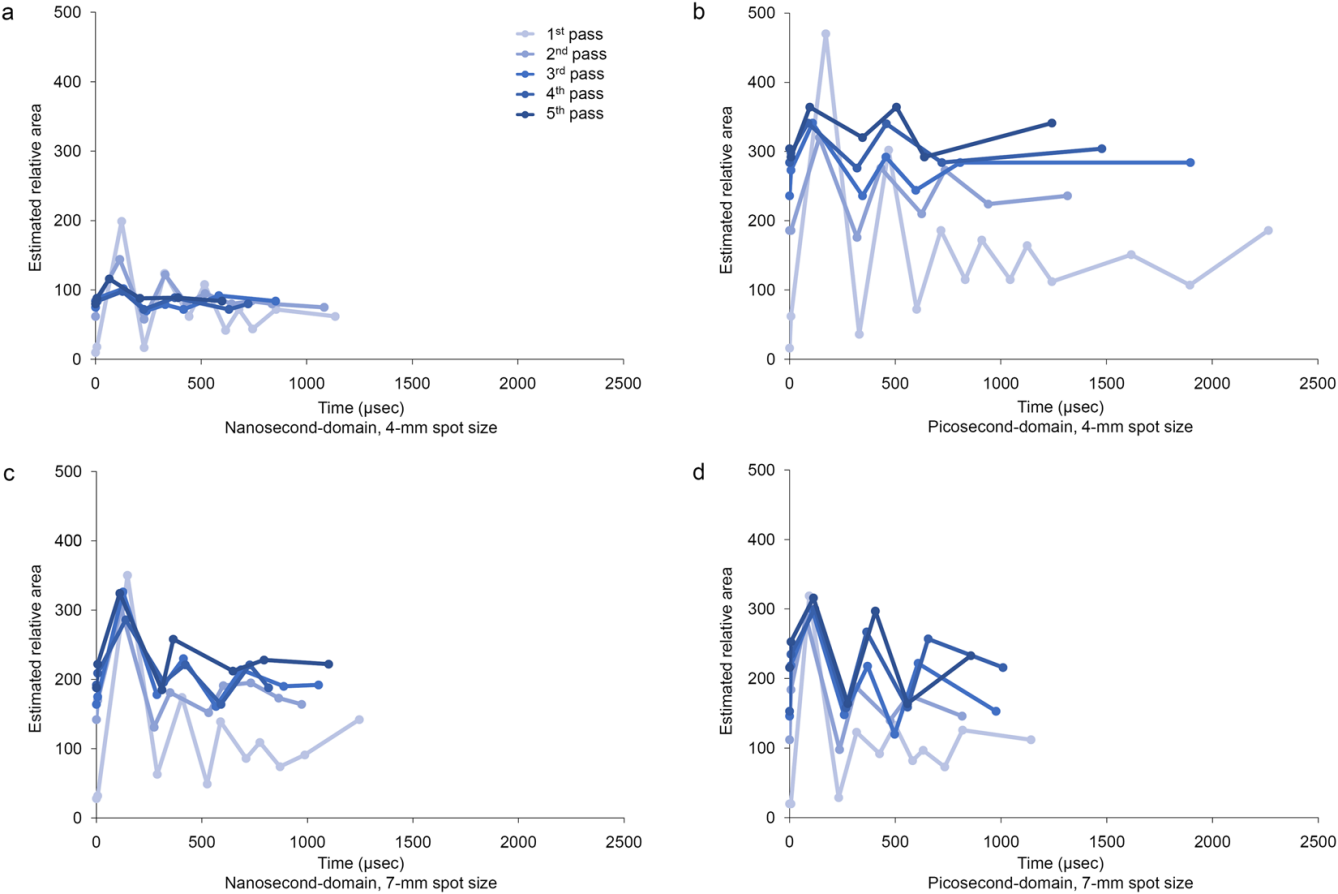
Time and estimated relative area of PIZs after Nd:YAG laser treatment. (**a**) Nanosecond-domain and (**b**) picosecond-domain Nd:YAG laser treatment at a 4-mm spot size and a laser fluence of 4.8 J/cm^2^. (**c**) Nanosecond-domain and (**d**) picosecond-domain Nd:YAG laser treatment at a 7-mm spot size and a laser fluence of 1.5 J/cm^2^.

Time-dependent estimation of the relative areas of PIZs indicated that the sizes of PIZs increased with repeated delivery of laser pulses in each experimental condition (Fig. 7). Furthermore, with repeated delivery of laser pulses, expansion and collapsing of the PIZs tended to reduce in magnitude for both the picosecond- and nanosecond-pulse duration lasers. Meanwhile, picosecond laser pulses generated more marked tissue reactions than nanosecond laser pulses, particularly at a 4-mm spot size.

## Discussion

In the present comparison study, we macroscopically analyzed laser-tattoo interactions generated by picosecond- and nanosecond-domain lasers in TM phantom. Tattoo pigment fractured into coarse particles after a single pulse of laser treatment, and continued delivery of laser energy further disintegrated the particles into smaller fragments that dispersed toward the boundaries of PIZs. Picosecond treatment broke the tattoo pigments down into more homogeneous particles than nanosecond treatment, and additional delivery of picosecond laser energy elicited further disintegration and dispersion of core tattoo particles into peripheral areas of PIZs, which was not achieved with additional nanosecond treatment.

In a previous simulation study on graphite tattoo particles, a strong acoustic wave was documented inside tattoo chromophores upon laser treatment, and the particles fractured once the tensile strength of the acoustic wave exceeded the strength threshold of the particle^5^. The study further demonstrated that a laser pulse duration shorter than the stress confinement time is more efficient at destroying tattoo particles^5^. A previous *in vivo* porcine model study also demonstrated that laser treatment at a wavelength of 758 nm and a pulse duration of 500 psec more effectively removes carbon tattoos than 755-nm Alexandrite laser treatment at a pulse duration of 30–50 nsec^10^. Taken together, these studies indicated that lasers with a picosecond-pulse duration may be more advantageous to the selective breakdown of tattoo particles than lasers with a nanosecond-pulse duration, which our results support.

Stress waves reflect and propagate inside individual ink particles from the edge thereof toward the center and the center toward the edge, and sufficient amplification of stress waves with which to cause fractures in graphite can be achieved in picoseconds^5^. In the present study, we found that, once individual ink particles had been fractured, repeated reflection and propagation of acoustic waves generated PIZs. These reactions were first observed within 6.51 µsec of treatment at a spot size of 4 mm using the picosecond-domain and nanosecond-domain laser, but not with a spot size of 7 mm. Prior to the development of irreversible PIZs, photoacoustic expansion was maximized during the first expansion and collapsing sequence and then gradually decreased over hundreds of microseconds to a few milliseconds, whereas collapsing was at a minimum at first and then increased. Interestingly, the estimated area of the initial macroscopic photoacoustic expansion was remarkably greater than the area of the stabilized PIZs. Accordingly, we suggest that the photoacoustic and photothermal effects of laser treatment on tattoo ink in the skin could extend beyond the expected boundaries of PIZs to surrounding tissue.

In this study, macroscopic patterns of interactions between laser energy and tattoo pigment particles were simulated using a TM phantom, which was mainly composed of polyacrylamide hydrogel and 5% (w/v) bovine serum albumin. A high concentration of acrylamide was used to heighten the attenuation coefficient, and bovine serum albumin was added as a temperature-sensitive indicator^13, 14^. A 5% (w/v) concentration of bovine serum albumin was chosen, and the TM phantoms in each experimental condition were prepared to the size of 1.0 cm × 1.0 cm × 2.5 cm. Although the pulse durations of the nanosecond- and picosecond-domain Nd:YAG lasers used in this study were 5 nsec and 750 psec, respectively, we found that residual light was observed at 6.51 µsec in most of the experimental settings. We discerned that the reflection and scattering of laser pulses in the limited space of the TM phantom may have contributed to the residual light.

TM phantoms are not identical to *in vivo* human tissue, which comprises a large number of factors that cause optical scattering and would theoretically decrease laser fluence and lengthen pulse duration on the way to target chromophores. This can cause picosecond energy to become nanosecond energy. Therefore, no scattering agent was added to the TM phantoms in our study to minimize optical scattering, allowing us to compare pulse duration-dependent laser-tattoo interactions. Accordingly, our simulation models using TM phantoms do not entirely reflect the intricate interactions of laser-tattoo interactions in *in vivo* human skin.

The maximum temperature inside irradiated tattoo ink particles is reportedly below the melting point of graphite, although sufficiently higher than the boiling point of water^5^. Thereby, although photothermal melting effects on tattoo ink cannot be obtained with laser treatment, cavitation bubbles are generated in the surrounding tissues^5^. In the present study, a tissue reaction mimicking vacuolization was also observed in the tissue surrounding the tattoo pigment after delivering the laser pulses. In the TM phantom models, cavitation bubbles were more readily apparent after treatment with the picosecond laser than the nanosecond laser. Lateral views of the treated TM phantoms revealed the formation of cavitation bubbles primarily at or above where the tattoo ink was inserted (baseline) upon treatment with the nanosecond laser, whereas they formed at or below the baseline upon treatment with the picosecond laser. High-speed cinematographs of picosecond laser treatment at a fluence of 4.8 J/cm^2^ and a spot size of 4 mm revealed a red-hot appearance at the bottom of PIZs, as well as cavitation bubbles. Thus, we propose that the shorter pulse duration of the picosecond-domain laser device could have more effectively generated boiling; however, the clinical significance of post-laser treatment vacuolization on tattoo removal remains to be elucidated, other than a known frosting appearance of the irradiated skin.

In conclusion, our macroscopic findings of laser-tattoo interactions demonstrated that treatment with picosecond- and nanosecond-domain lasers results in a characteristic feature of PIZ formation. While simulation models using TM phantoms do not entirely reflect the intricate interactions among laser pulses, tattoo particles, cellular components, and adnexal structures, our comparative study, nonetheless, demonstrated that picosecond treatment breaks tattoo pigments down more homogeneously than nanosecond treatment. Moreover, additional delivery of picosecond laser energy facilitated further disintegration and dispersion of core tattoo particles into peripheral areas of PIZs. Finally, high-speed cinematography revealed that PIZs form after repeated courses of acoustic wave reflection and propagation over hundreds of microseconds to a few milliseconds. Although further comparative study using *in vivo* human skin is needed, we believe that our findings may help with predicting three-dimensional laser-tattoo interactions and associated reactions in surrounding tissues.

## Methods

### Tissue-mimicking phantom and tattoo pigment

The polyacrylamide hydrogel TM phantom was prepared by mixing 5% (w/v) bovine serum albumin (Sigma-Aldrich, St. Louis, MO, USA) in distilled water according to a previous report with minor modification^13, 14^. After degassing, a 25% (v/v) aqueous solution of 40% (w/v) acrylamide (Sigma-Aldrich) was added to the mixture. Then, polymerization was initiated by adding 10% (v/v) of 1 mol/L of TRIS buffer at a pH of 8 (Sigma-Aldrich) and 0.84% (v/v) of a 10% (w/v) ammonium persulfate solution (Sigma-Aldrich). After an additional degassing, 0.05% (v/v) of N, N, N’, N’-tetramethyl ethylenediamine (Sigma-Aldrich) was added for polymerization. The final mixture was then immediately poured into a 1.0 cm × 1.0 cm × 2.5 cm rectangular polycarbonate housing and solidified in a refrigerator at 4 °C. Then, 0.02 ml of black tattoo ink (Intenze; South Rochelle Park, NJ, USA) was gently inserted in the TM phantom using a 30-gauge, 2.5-mm needle on a 1-ml syringe.

### Laser devices and treatment settings

A picosecond-domain 1,064-nm Nd:YAG laser (PICO^+^4^TM^; Lutronic Corp., Goyang, Korea) and a nanosecond-domain 1,064-nm Nd:YAG laser (SPECTRA XT^TM^; Lutronic Corp.) were used in this study. TM phantoms with black tattoo ink were treated with the picosecond-domain Nd:YAG laser at a pulse duration of 750 psec and the nanosecond-domain Nd:YAG laser at a pulse duration of 5 nsec. Laser fluences of 1.8 J/cm^2^, 2.8 J/cm^2^, 3.8 J/cm^2^, and 4.8 J/cm^2^ at a spot size of 4 mm and fluences of 0.5 J/cm^2^, 0.7 J/cm^2^, 1.0 J/cm^2^, 1.2 J/cm^2^, and 1.5 J/cm^2^ at a spot size of 7 mm were delivered to the TM phantoms using both the picosecond- and nanosecond-domain Nd:YAG lasers.

In additional experiments, five passes of laser treatment using the nanosecond-domain Nd:YAG laser at a pulse duration of 5 nsec were initially delivered to TM phantoms to disintegrate ink particles. This was followed by five passes with the nanosecond- or picosecond-domain Nd:YAG laser at fluences of 4.8 J/cm^2^ for a 4-mm spot size and 1.5 J/cm^2^ for a 7-mm spot size. Similarly, five passes of laser treatment using the picosecond-domain Nd:YAG laser at a pulse duration of 750  the same fluences and spot sizes listed above. Immediately after delivering the five initial passes to the TM phantom and after the five final passes in each experimental setting, photographs were taken using a digital single-lens reflex camera (Nikon D90; Nikon Corp., Tokyo, Japan) with a micro Nikkor AF-S VR lens (Nikon Corp.) at a focal length of 105 mm and f/2.8G internal focusing-extra-low dispersion. All experiments were performed in triplet. To help visualize differences in laser-induced tattoo particle fragmentation, all images were converted to an 8-bit gray scale and, then, adjusted according to the histogram RGB settings of 0–50 using Image-Pro Plus software, version 7.0 (Media Cybernetics, Silver Spring, MD, USA).

### High-speed cinematography

To visualize laser-tattoo particle interactions within the TM phantom, a high-speed digital video camera (Phantom V2512; Vision Research Inc., Wayne, NJ, USA) under two light-emitting diode spotlights (120 W) was utilized at a capture rate of 460,000 frames per second over a period of 2.17 µs and a resolution of 128 × 128 pixels. Video footage was recorded from the moment of laser irradiation until PIZ formation. In these experiments, TM phantoms were treated with a laser fluence of 4.8 J/cm^2^ at a spot size of 4 mm and a fluence of 1.5 J/cm^2^ at a spot size of 7 mm using the picosecond- and nanosecond-domain 1,064-nm Nd:YAG lasers. Five pulses thereof were applied at a frequency of 5 Hz for optimized visualization. Representative frames at baseline and immediately after irradiation, as well as upon maximum expansion and minimum collapsing of PIZs, in each experimental setting were captured using Adobe Premiere Pro CC 2015, version 9.0 (Adobe Systems, San Jose, CA, USA). The areas of representative PIZs were measured using Image J software, version 1.48 (National Institutes of Health, Bethesda, MD, USA).

## Electronic supplementary material

Supplementary Information

Supplementary video 1

Supplementary video 2

Supplementary video 3

Supplementary video 4
