# Supplementary material for: Pattern analysis of laser-tattoo interactions for picosecond- and nanosecond-domain 1,064-nm neodymium-doped yttrium-aluminum-garnet lasers in tissue-mimicking phantom
Source: Sci Rep. 2017 May 8;7:1533. doi: 10.1038/s41598-017-01724-1 (PMC5431496; doi:10.1038/s41598-017-01724-1)
Supplement: Supplementary file 1 — Supplementary Information [file 41598_2017_1724_MOESM1_ESM.pdf]

ARTICLE

Pattern analysis of laser-tattoo interactions for picosecond- and nanosecond-domain 1,064-nm neodymium-doped yttrium-aluminum-garnet lasers in tissue-mimicking phantom

Keun Jae Ahn[1], Zhenlong Zheng[2], Tae Rin Kwon[3,4], Beom Joon Kim[3,4], Hye Sun Lee[5] & Sung Bin Cho[3,6,7]

[1]Department of Science Education, Jeju National University, Jeju, Korea; [2]Department of Dermatology, Yanbian University Hospital, Yanji, China; [3]Department of Dermatology, Chung-Ang University College of Medicine, Seoul, Korea; [4]Department of Medicine, Graduate School, Chung-Ang University, Seoul, Korea; [5]Biostatistics Collaboration Unit, Yonsei University College of Medicine, Seoul, Korea; [6]Department of Dermatology, International St. Mary's Hospital, Catholic Kwandong University, College of Medicine, Incheon, Korea; [7]Kangskin Sillim Dermatology Clinic, Seoul, Korea.

Correspondence and requests for samples should be addressed to Sung Bin Cho, MD, PhD, Department of Dermatology, Chung-Ang University Hospital 102 Heukseok-ro, Dongjak-gu, 156-755 Seoul, Korea. Tel.: +82.2.6299-3081, Fax: +82.2.811-1159. E-mail: drsbcho@gmail.com.

Total word count: 3,223
Number of references: 14
Number of tables: 0
Number of figures: 7
Number of supplementary figures: 2
Number of supplementary videos: 4
Conflicts of interest: None declared
Funding sources: None
Running Heads: PICOSECOND VERSUS NANOSECOND LASERS
Keywords: Laser, acoustic wave, neodymium-doped yttrium aluminum garnet, pulse duration, picosecond, nanosecond, tattoo pigment, tissue-mimicking phantom

SUPPLEMENTARY FIGURES

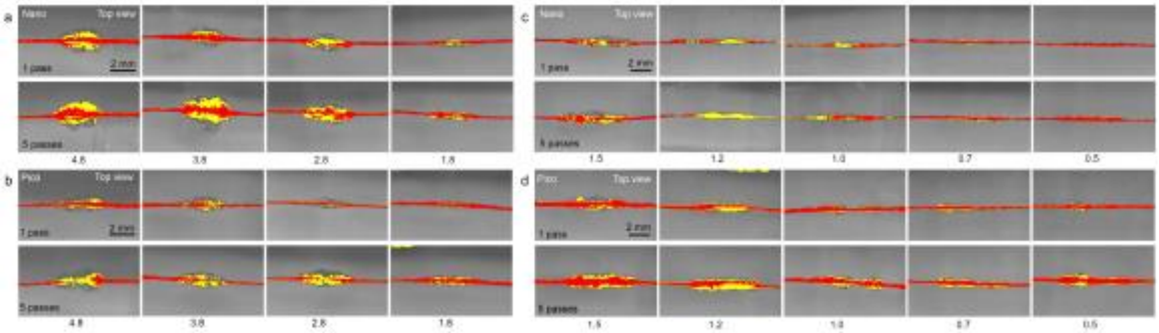

Supplementary figure 1. Adjusted images after picosecond- and nanosecond-domain neodymium-doped yttrium aluminum garnet (Nd:YAG) laser treatments on tattoo ink embedded in tissue-mimicking (TM) phantom. Both the (**a**) nanosecond- and (**b**) picosecond-domain Nd:YAG laser treatments generated cocoon-shaped or oval photothermal and photoacoustic injury zones (PIZ) at the laser fluences of 4.8 J/cm^2, 3.8 J/cm^2, 2.8 J/cm^2, and 1.8 J/cm^2 and at a spot size of 4 mm. At laser fluences of 1.5 J/cm^2, 1.2 J/cm^2, 1.0 J/cm^2, 0.7 J/cm^2, and 0.5 J/cm^2 and a spot size of 7 mm, cocoon-shaped PIZs formed after the (**c**) nanosecond- and (**d**) picosecond-domain Nd:YAG laser treatments. Top view. Laser fluences are presented as J/cm^2. Nano, nanosecond-domain laser; Pico, picosecond-domain laser.

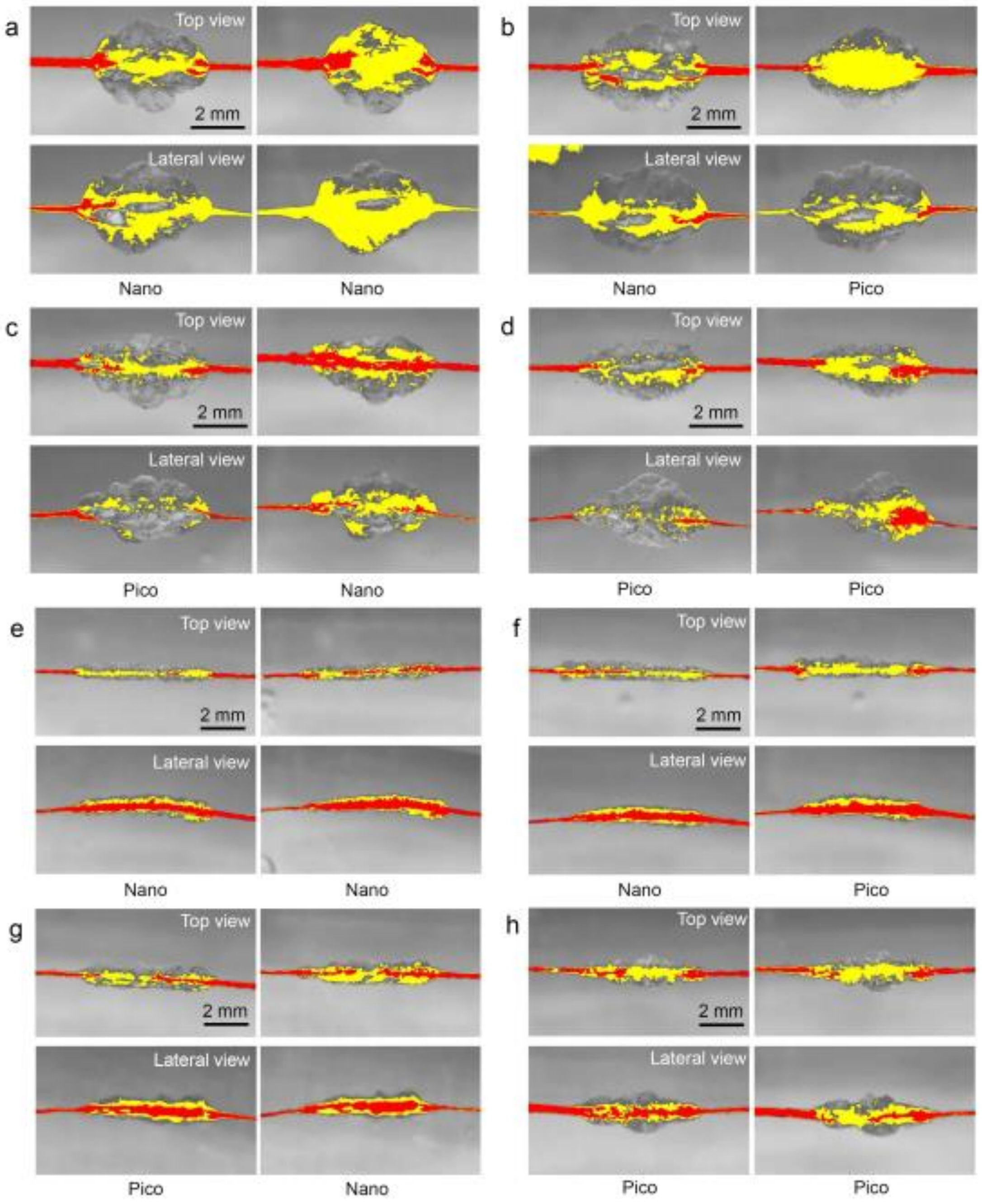

Supplementary figure 2. Adjusted images after combination of picosecond- and nanosecond-domain Nd:YAG laser treatment on tattoo ink embedded in TM phantom. Five passes of laser treatment using each of the picosecond- and nanosecond-domain Nd:YAG lasers were initially performed to fracture the ink particles. Then, an additional five passes with either the

nanosecond- or picosecond-domain Nd:YAG laser were delivered. (**a**) Nanosecond-then-nanosecond, (**b**) nanosecond-then-picosecond, (**c**) picosecond-then-nanosecond, and (**d**) picosecond-then-picosecond at the experimental settings of a 4-mm spot size and a laser fluence of 4.8 J/cm^2. (**e**) Nanosecond-then-nanosecond, (**f**) nanosecond-then-picosecond, (**g**) picosecond-then-nanosecond, and (**h**) picosecond-then-picosecond at the experimental settings of a 7-mm spot size and a laser fluence of 1.5 J/cm^2. Top and lateral views. Nano, nanosecond-domain laser; Pico, picosecond-domain laser.

SUPPLEMENTARY VIDEO LEGENDS

Supplementary video 1. High-speed cinematography of nanosecond-domain neodymium-doped yttrium aluminum garnet (Nd:YAG) laser treatment on tattoo-embedded TM phantom. Immediately after the first pulse of nanosecond-domain Nd:YAG laser treatment at the laser fluence of 4.8 J/cm^2 and a spot size of 4 mm. Lateral view.

Supplementary video 2. High-speed cinematography of picosecond-domain Nd:YAG laser treatment on tattoo-embedded TM phantom. Immediately after the first pulse of picosecond-domain Nd:YAG laser treatment at the laser fluence of 4.8 J/cm^2 and a spot size of 4 mm. Lateral view.

Supplementary video 3. High-speed cinematography of nanosecond-domain Nd:YAG laser treatment on tattoo-embedded TM phantom. Immediately after the first pulse of nanosecond-domain Nd:YAG laser treatment at the laser fluence of 1.5 J/cm^2 and a spot size of 7 mm. Lateral view.

Supplementary video 4. High-speed cinematography of picosecond-domain Nd:YAG laser treatment on tattoo-embedded TM phantom. Immediately after the first pulse of picosecond-

domain Nd:YAG laser treatment at the laser fluence of 1.5 J/cm^2 and a spot size of 7 mm. Lateral view.
